# Metabolomic Study of Zuojin Pill in Relieving 1-Methyl-3-nitro-1-nitrosoguanidine-Induced Chronic Atrophic Gastritis

**DOI:** 10.1155/2021/7004798

**Published:** 2021-12-17

**Authors:** Yuling Tong, Manyi Jing, Xu Zhao, Honghong Liu, Shizhang Wei, Ruisheng Li, Xia Liu, Min Wang, Hongtao Song, Yanling Zhao

**Affiliations:** ^1^Department of Pharmacy, Chinese PLA General Hospital, Beijing, China; ^2^College of Pharmacy, Chengdu University of Traditional Chinese Medicine, Chengdu, China; ^3^Senior Department of Hepatology, The Fifth Medical Center of PLA General Hospital, Beijing, China; ^4^Research Center for Clinical and Translational Medicine, Fifth Medical Center of PLA General Hospital of Chinese, Beijing, China; ^5^Department of Pharmacy, 900 Hospital of the Joint Logistics Team, Fuzhou, China

## Abstract

The classic prescription Zuojin Pill (ZJP) shows a good therapeutic effect on chronic atrophic gastritis (CAG); it is of great significance to clarify its specific mechanism. Therefore, we explore the mechanism of ZJP on MNNG-induced CAG by integrating approaches. First of all, through the pathological changes of gastric tissue and the expression level of PGI and PGI/II in serum, the expression of inflammation-related factors was determined by RT-PCR to determine the efficacy. Then, UPLC-Q-TOF/MS was used for plasma and urine metabolomic analysis to screen the specific potential biomarkers and metabolic pathway of ZJP in ameliorating CAG and to explore its possible mechanism. ZJP significantly ameliorate the pathological injury of gastric tissue, increase levels of PGI and PGI/II, and reduce the expression level of proinflammatory factors. Through metabolomic analysis, 9 potential metabolic differences were identified and 6 related metabolic pathways were enriched. These findings indicate for the first time the potential mechanism of ZJP in improving CAG induced by MNNG and are of great significance to the clinical development and application of ZJP-related drugs.

## 1. Introduction

Gastric cancer (GC) is the fourth most common cancer and one of the leading causes of cancer-related death in the world [1]. Chronic atrophic gastritis (CAG) is characterized by inflammation and gastric gland structure loss [2]. Gastric mucosal atrophy is a key stage in the progression of GC, so paying attention to the prevention and treatment of CAG can effectively reduce the incidence of GC [3]. 1-Methyl-3-nitro-1-nitrosoguanidine (MNNG) can cause base mutation on DNA and has strong carcinogenicity. MNNG can be used to simulate improper nitrate intake and is often used to establish the CAG model.

ZJP is composed of *Coptis chinensis* Franch. and *Tetradium ruticarpum* (A.Juss.) T.G.Hartley at 6 : 1. Both are first written in Shennong Materia Medica Sutra. *Coptis chinensis* has bitter taste, cold nature, purging fire, detoxification, and clearing heat. *Tetradium ruticarpum* (A.Juss.) T.G.Hartley has the effect of pungent, bitter, hot, antinausea, dispelling cold, and relieving pain. The 2020 edition of Chinese Pharmacopoeia records that *Coptis chinensis*, processed with *Tetradium ruticarpum* (A.Juss.) T.G.Hartley juice, can relieve liver and stomach and stop vomiting. It is used for disharmony between the liver and stomach, vomiting, and swallowing acid. Recent research studies have shown that ZJP has a good therapeutic effect on gastric ulcer [4], ulcerative colitis [5], and other diseases. A meta-analysis based on a systematic review highlights that ZJP is effective and safe for chronic gastritis [6]. Our previous studies [7] showed that ZJP could ameliorate the injury of GES-1 and MNNG-induced CAG rats. However, the specific mechanism is not clear.

Metabolomics, which aims to reveal the metabolites and a thorough snapshot of various metabolic processes in biological systems, is a new field of “omic” science in systems biology [8]. Metabolomics can evaluate the effect changes caused by diet, lifestyle, and environmental factors, which is a key tool for biomarker discovery [9]. Metabolome and related pathways also help us to understand the pathophysiology and pathogenesis of diseases. Blood and urine are two biofluids that are frequently sampled and analyzed to obtain relevant information. In this study, plasma and urine were used for metabolomic analysis to explore the ameliorative effect of ZJP on CAG and the characteristics of body metabolites, so as to provide great inspiration for elucidating the mechanism.

## 2. Materials and Methods

### 2.1. Reagents

Vitacoenzyme tablets (lot no. 200204) were purchased from Guangxi Dahai Sunshine Pharmaceutical Co., Ltd. MNNG (purity ≥95%, CAS: 70-25-7) was supplied by Aladdin Biochemical Technology Co., Ltd. (Shanghai, China). Enzyme-linked immunosorbent assay (ELISA) kits for interleukin (IL)-1*β*, interleukin (IL)-6, pepsinogen I (PGI), and pepsinogen II (PGII) were obtained from Shanghai Enzyme-Linked Biology Co., Ltd. Tissue RNA purification kit plus (Cat. no. RN002 plus) was purchased from Yishan Biotechnology Co., Ltd. (Beijing, China). RevertAid First Strand cDNA Synthesis Kit (lot: 00976964) was provided by Thermo Fisher Scientific (USA).

### 2.2. ZJP Preparations

*Coptis chinensis* (lot: 18011901) and *Tetradium ruticarpum* (A.Juss.) T.G.Hartley (lot: 19071501) were purchased from Lvye Pharmaceutical Co., Ltd. (Beijing, China). *Coptis chinensis* (inspection report number: CP-18-01-22) and *Tetradium ruticarpum* (A.Juss.) T.G.Hartley (CP-19-07-09) were tested by high performance liquid chromatography (HPLC) and thin layer chromatography (TLC), and the quality conformed to the 2015 edition of Chinese Pharmacopoeia. Weighing *Coptis chinensis* and *Tetradium ruticarpum* (A.Juss.) T.G.Hartley at 6 : 1 was extracted twice by heating (1/10, w/v; 1 h/one time). The filtrate was rotationally evaporated, condensed, and dried into dry powder for 4°C preservation, and the final mass ratio of ZJP was 23.4%. Its high performance liquid phase analysis has been carried out in previous studies [7].

### 2.3. Animal Handling

Male SPF Sprague Dawley rats (150 g-170 g; SCXK-(jing) 2019-0010) were purchased from SiPeiFu Animal Breeding Center (Beijing, China). Adaptive feeding was followed for one week under the conditions of 25 ± 0.5°C, 55 ± 5%. 48 rats were randomly divided into the control group, MNNG group, vitamin tablets group (200 mg/kg), and ZJP high-dose (2.52 g/kg), medium-dose (1.26 g/kg), and low-dose (0.63 g/kg) group (*n* = 8). Except for the control group, the other groups were given free drinking of MNNG (170 ug/mL). MNNG was given intragastrically every other day (170 ug/mL, 1 mL/100 g). In addition, adequate feeding was followed for one day, fasting for one day, and so repeatedly for 10 weeks. Then, the corresponding drugs were given by gavage for 4 weeks (1 mL/100 g, 1/d). 12 hours after the last administration, the rats were put into metabolic cages for urine collection, 1 rat in each cage, gave water but no food, and collected for 12 hours. The blood was collected from the abdominal aorta and collected by collecting blood vessels containing EDTA-k2. After being placed for 2 h, blood was centrifuged at 4°C, 3000 rpm for 15 min, and the supernatant was collected for reserve.

### 2.4. Pharmacodynamic Index Detection

The expression level of IL-6, IL-1 *β*, PGI, and PGII in the collected serum samples was detected by ELISA kit. Hematoxylin-eosin (H & E) staining was used to observe the pathological changes of gastric tissue.

### 2.5. Quantitative RT-PCR

According to the instructions, tissue RNA purification kit plus and reverse transcription kit were used to extract total RNA of stomach tissue and reverse transcribed to cDNA. The data were analyzed by the 2^−ΔΔCT^ method, and the information of primers is given in Table 1.

### 2.6. Sample Preparation and Detection

Plasma or urine after 200 ul remelting was mixed with 600 ul methanol, centrifuged at 4°C and 12000 rpm for 10 minutes. The supernatant was collected and filtered with 0.22 *μ*m membrane to get the sample to be tested. The plasma and urine samples were measured on an Agilent 6550A Q-TOF/MS instrument (Agilent Technologies, Santa Clara, USA) and ZORBAX RRHD 300 SB-C18 column (2.1 mm × 100 mm, 1.8 *μ*m, Agilent Technologies, Santa Clara, USA) for chromatography and separation. The setting of relevant parameters has been reported in our previous research [10].

### 2.7. Data Process and Multivariate Analysis

First of all, use MassHunter Profinder software (version B.06.00, Agilent, California, USA) was used to extract sample data. The extracted data are normalized in MetaboAnalyst 5.0. Next, the normalized data are imported into SIMCA (version 14.1, MKS Umetrics), and principal component analysis (PCA) and orthogonal partial least square discriminant analysis (OPLS-DA) are carried out, respectively [11]. Select metabolic samples with |P(corr)| ≥0.58 and VIP >1 for further analysis. The METLIN (http://metlin.scripps.edu/) database and Human Metabolome Database (HMDB) (https://www.hmdb.ca/) were used to identify potential biomarkers. Potential markers visualized the enrichment pathway by MetaboAnalyst 5.0.

### 2.8. Statistics Analysis

All data were analyzed with the SPSS 17.0 (Chicago, IL, USA) and presented as mean ± standard deviation (SD). One-way analysis of variance (ANOVA) was for data analysis. *P* < 0.01 was highly significant and *P* < 0.05 was considered statistically significant. GraphPad Prism 8 (San Diego, USA) was utilized for visible presentation of all results.

## 3. Results

### 3.1. Relieving Pathological Injury of CAG

**Figure 1:**
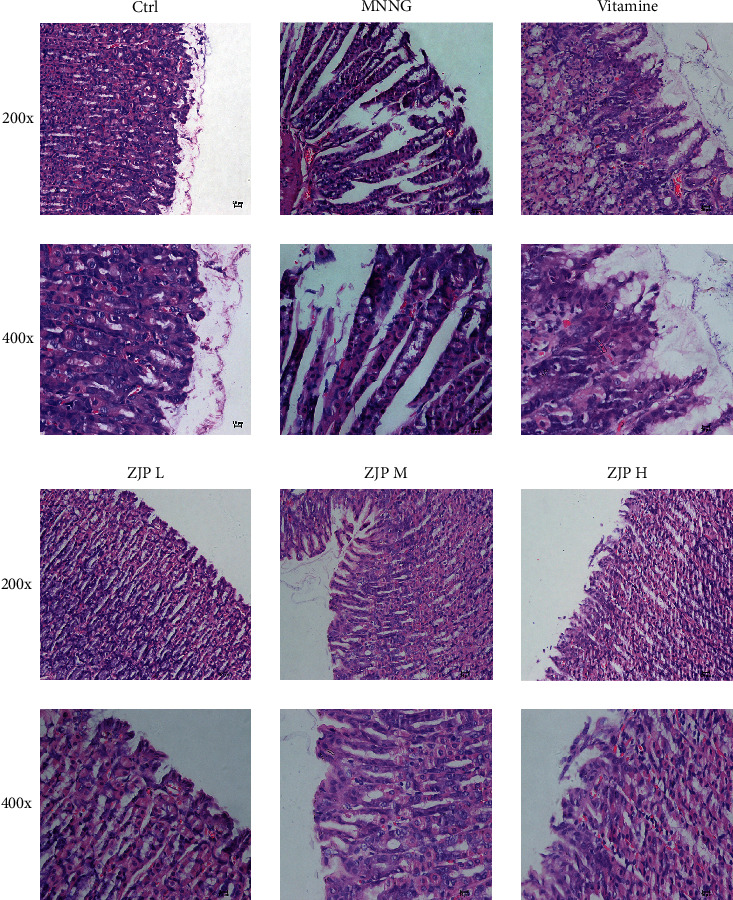
Pathological changes of gastric mucosa in CAG rats.

As shown in Figure 1, compared with the control group, the gastric mucosa became thinner, the glandular gastric mucosal epithelial cells atrophied severely, the gastric glandular middle wall cells atrophied, and the submucosae inflammatory cells infiltrated in the MNNG group. Intervention of ZJP (especially the high dose) can improve the atrophy of inherent glands of gastric mucosa and reduce the infiltration of inflammatory cells.

### 3.2. Improve Serum Biochemical Indexes

**Figure 2:**
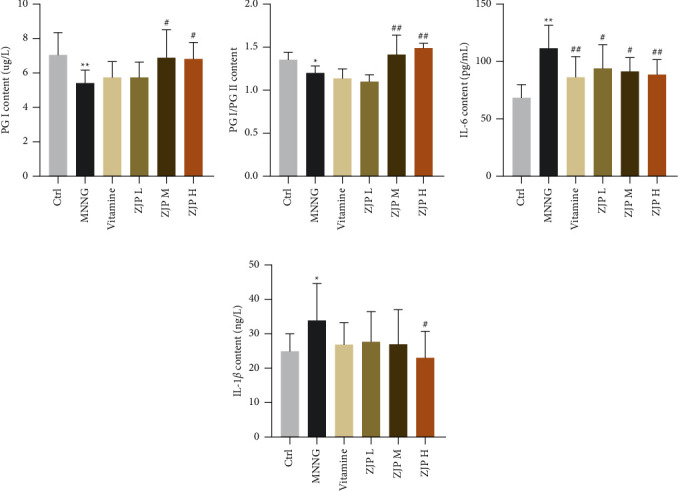
Detection of PGI, PG, IL-6, and IL-1*β* expression in serum of CAG rats by ELISA kit. ^*∗∗*^<0.01 and ^*∗*^<0.05 versus the control group; ^##^<0.01 and ^#^<0.05 versus the MNNG group. Data are expressed as mean ± SD (*N* = 8).

The expression levels of IL-6, IL-1*β*, PGI, and PGII in serum of rats were detected by ELISA kit. The results showed that the contents of PGI and PGI/PG in the MNNG group were significantly lower (*P* < 0.01 or *P* < 0.05), while the expression of IL-1*β* and IL-6 was significantly higher than the control group (*P* < 0.01 or *P* < 0.05) (Figure 2). Compared with the MNNG group, the level of PGI and the ratio of PGI/PGII in the middle and high-dose groups of ZIP increased significantly (*P* < 0.01 or *P* < 0.05). The expression of IL-6 decreased significantly in the vitamin group and three dose groups of ZJP (*P* < 0.01 or *P* < 0.05). The expression of IL-1*β* was significantly decreased in the high-dose group of ZJP (*P* < 0.05) and tended to decrease in other dose groups, but there was no significant difference (*P* > 0.05).

### 3.3. Reduce the Expression of Inflammatory Factors

**Figure 3:**
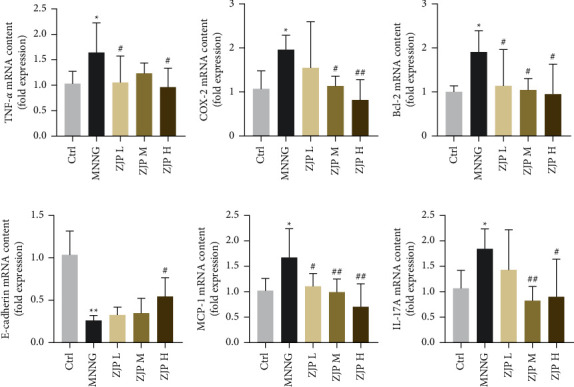
Expression of related mRNA. (a) TNF-*α*. (b) COX-2. (c) Bcl-2. (d) E-cadherin. (e) MCP-1. (f) IL-17A. ^*∗∗*^<0.01 and ^*∗*^<0.05 versus the control group; ^##^<0.01 and ^#^<0.05 versus the MNNG group. Data were expressed as mean ± SD (*N* = 8).

In the MNNG group, the expression of cyclooxygenase-2 (COX-2), tumor necrosis factor (TNF)-*α*, interleukin (IL)-17A, B cell lymphoma-2 (Bcl-2), and monocyte chemoattractant protein (MCP)-1 in gastric tissue was significantly higher than that of the control group (*P* < 0.05). The expression of COX-2, TNF-*α*, IL-17A, Bcl-2, and MCP-1 was significantly decreased after the intervention of middle and high-dose of ZJP (*P* < 0.01 or *P* < 0.05), and the expression of TNF-*α*, Bcl-2, and MCP-1 was significantly decreased in the low-dose group of ZJP (*P* < 0.05). In addition, the expression of E-cadherin mRNA was significantly decreased in the MNNG group (*P* < 0.01) and was significantly increased in the high-dose ZJP group (*P* < 0.05) (Figure 3).

### 3.4. Multivariate Statistical Analysis

**Figure 4:**
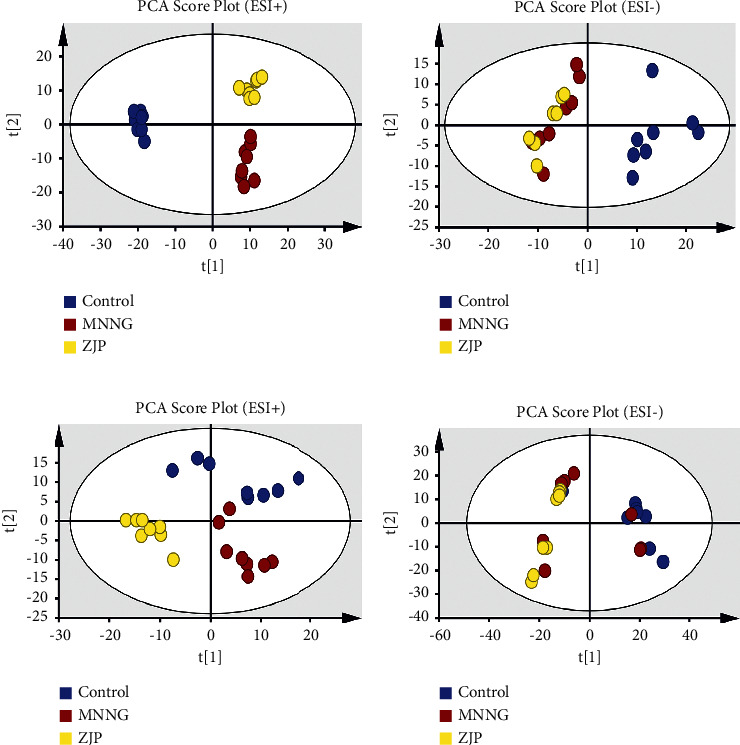
PCA score plot. (a)-(b) Plasma; (c)-(d) urine.

**Figure 5:**
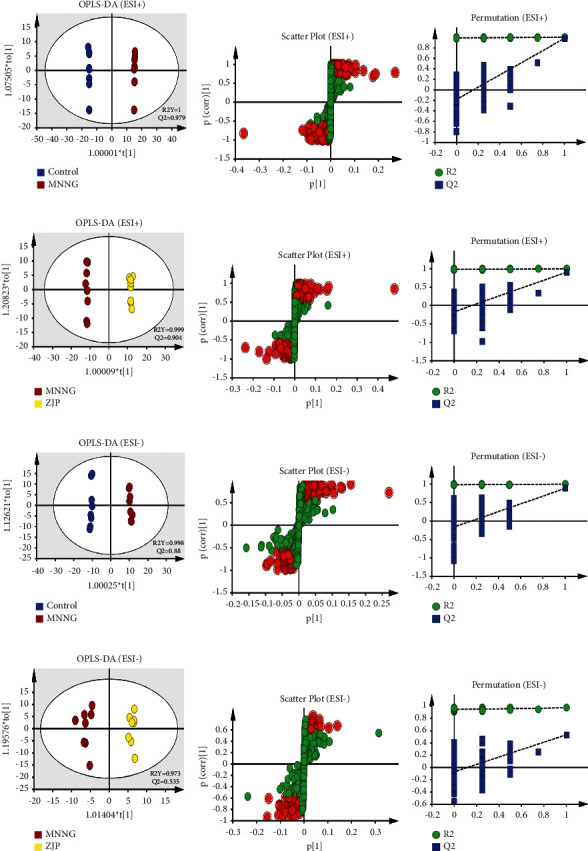
The OPLS-DA plots (a), (d), (g), (j), S-plot (b), (e), (h), (k), and 100-permutation test (c), (f), (i), (l) of plasma metabolomics analysis.

**Figure 6:**
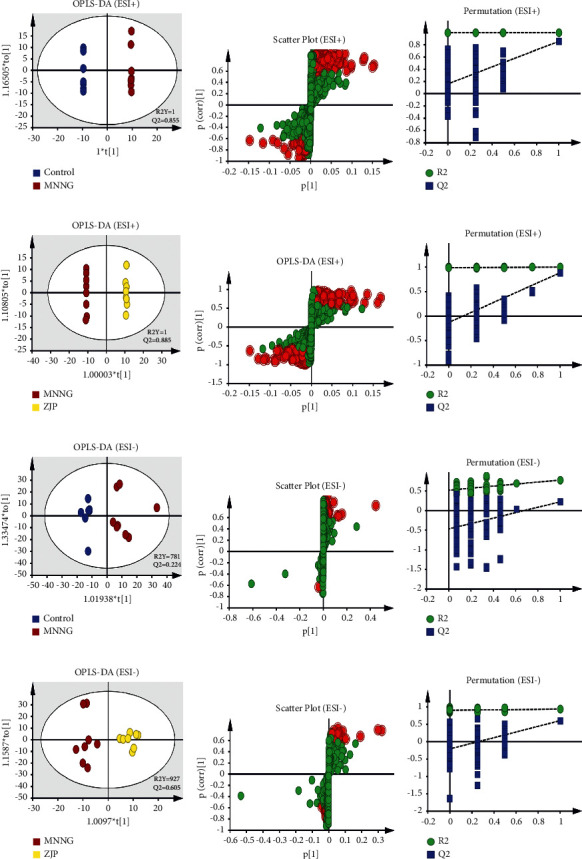
The OPLS-DA plots of (a), (d), (g), (j), S-plot (b), (e), (h), (k)), and 100-permutation test (c), (f), (i), (l) of urine metabolomics analysis.

PCA is an unsupervised data mining method and the basis of multivariate analysis. Results of PCA score map showed that there was a certain trend of separation among different groups (Figure 4). Furthermore, the supervised analysis method, OPLS-DA, was used to analyze the metabolism of plasma and urine between the control group and the MNNG group and the MNNG group and the ZJP group to find out the different variables. It was observed that there was a good trend of separation between different groups, suggesting that there were different metabolites (Figures 5 and 6). The variables with VIP >1, | Pcorr | >0.58 were selected as potential differential metabolites, and the metabolites coexpressed were further analyzed.

### 3.5. Biomarker Screenings and Pathway Enrichment Analysis

**Figure 7:**
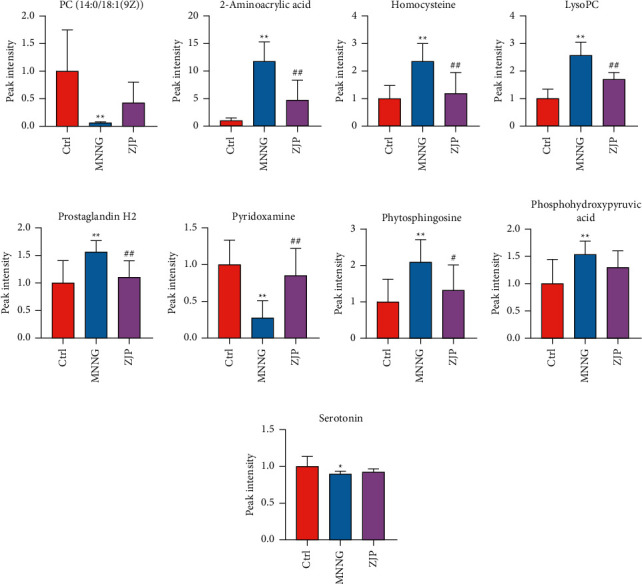
Changes of potential differential metabolites. (a) PC (14 : 0/18 : 1(9Z)). (b) 2-Aminoacrylic acid. (c) Homocysteine. (d) LysoPC (20 : 5(5Z,8Z,11Z,14Z,17Z)/0 : 0). (e) Prostaglandin H2. (f) Pyridoxamine. (g) Phytosphingosine. (h) Phosphohydroxypyruvic acid. (i) Serotonin. Data are shown as mean ± SD (*N* = 8). ^*∗∗*^<0.01 versus the control group; ^*∗*^<0.05 versus the control group; ^##^ <0.01 versus the MNNG group; ^#^<0.05 versus the MNNG group. Ctrl, control.

**Figure 8:**
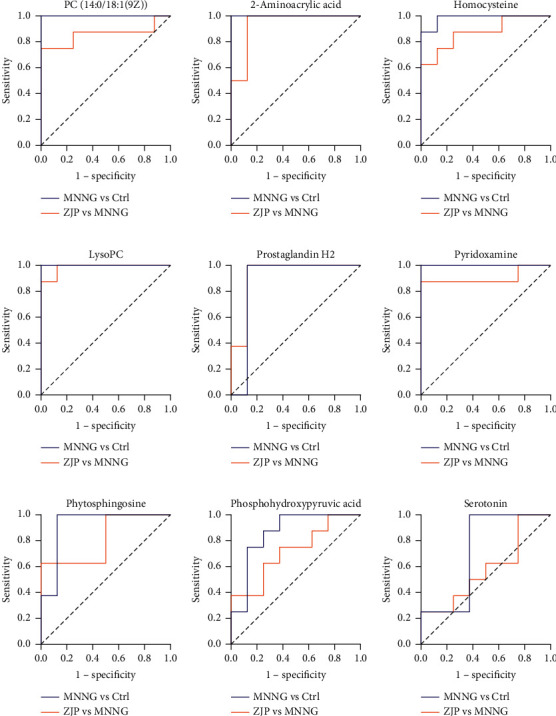
The area under the ROC curve based on the potential biomarkers.

**Figure 9:**
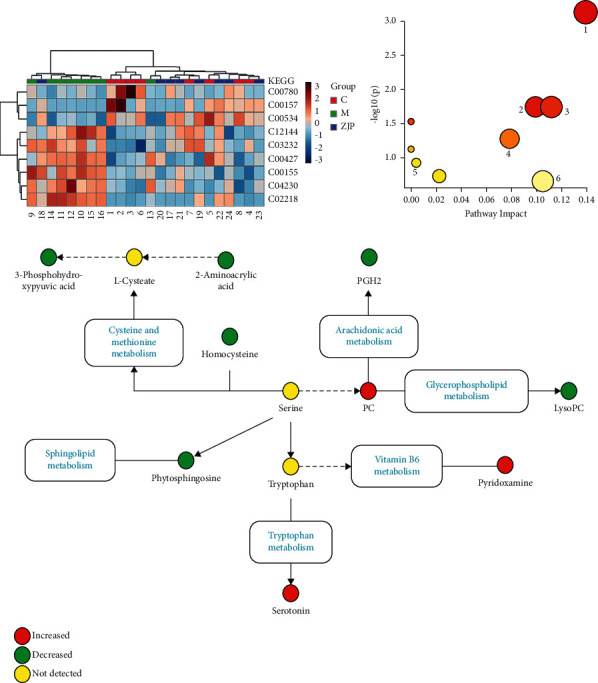
Enrichment analysis of metabolic markers. (a) The heatmap of potential metabolites. (b) The metabolic pathways involved in the therapeutic effects of ZJP on CAG. (c) Relationship among the metabolic biomarkers and metabolic pathways. Red circle indicates peak area ZJP/MNNG >1, and green circle indicates peak area ZJP/MNNG <1.

The charge-mass ratio of the selected coexpressed metabolites was substituted into HMDB and METLIN data base to obtain the corresponding compounds. The marker metabolites were selected according to *P* < 0.05. The basic characteristics of these metabolites are given in Table 2 and Figure 7. We plotted the area under the receiver operating characteristic (ROC) curve (AURC) to judge the discriminant ability and reliability of the selected biomarkers. The results showed that except for the low accuracy of serotonin, the AURC values of other markers were greater than 0.8 (Figure 8), suggesting that the metabolites we screened were iconic. Considering the structure of the pathway and visualizing the dynamic pathway are helpful to enhance the enrichment analysis of set metabolites. MetaboAnalyst 5.0 was used for visual analysis and enrichment analysis of iconic metabolites, and the cluster analysis heat map reflected the differences in the expression of metabolites among groups (Figure 9(a)). Pathway enrichment analysis is given in Table 3 and Figures 9(b) and 9(c), and the main metabolic pathways involved include the cysteine and methionine metabolism, sphingolipid metabolism, arachidonic acid metabolism, glycerophospholipid metabolism, vitamin B6 metabolism, and tryptophan metabolism.

## 4. Discussion

2019 updated management of precancerous conditions and lesions in the stomach guide [12] mentions that low PG I serum levels or low PG I/ratio can be used as good indicators of gastric mucosal atrophy. Our results showed that PGI and PGI/decreased significantly in the MNNG group, suggesting that gastric mucosal atrophy occurred in CAG rats. ZJP intervention could reverse this change in varying degrees. The progression of CAG induces the secretion of proinflammatory factors, which in turn amplify gastric mucosal injury. TNF-*α* is an important proinflammatory factor from macrophages in the process of gastric inflammation and carcinogenesis [13, 14]. COX catalyzes arachidonic acid biosynthesis of prostaglandins, in which the overexpression of COX-2 is closely related to the severity of gastritis [15]. IL-1*β* induces the expression of cytokines such as IL-6 and COX-2 to aggravate the inflammatory response and further lead to gastric mucosal injury. Wang and Chang [16] found that the expression of IL-1*β*, IL-6, TNF-*α*, and COX-2 increased significantly in gastric precancerous lesion model rats. Our results showed that the expressions of inflammatory cytokines such as IL-1*β*, IL-6, TNF-*α*, COX-2, and MCP-1 were significantly increased in CAG rats, suggesting that the progression of CAG is closely related to inflammation. After intervention with ZJP, it can significantly reduce the expression level of proinflammatory factors and inflammatory infiltration. E-cadherin is an important adhesion molecule in epithelial tissue to maintain cellular structure, and its loss of function is often closely related to gastric mucosal injury [17]. IL-17 can induce ROS production and trigger parietal cell atrophy and death in inflammatory microenvironment [18]. In our results, the expression of E-cadherin was decreased, and IL-17 was significantly overexpressed in CAG rats. In the treatment of ZJP, the high-dose group has the best effect, so the high-dose group is selected for further metabolic analysis. The results indicate that ZJP can improve gastric mucosal atrophy, restore gastric mucosal function, and reduce inflammation.

In our further research, through the screening and enrichment analysis of differential metabolites in plasma and urine, we obtained 9 potential metabolic differences and enriched 6 related metabolic pathways. After the intervention of ZJP, the expression of metabolites returned to the normal level.

Excessive reactive oxygen species (ROS) can cause tissue damage and chronic inflammation. Cysteine and methionine play an important role in regulating immune system function and reducing oxidative stress through their metabolites [19]. Arachidonic acid can participate in and promote a variety of inflammatory-related responses and is an important inflammatory mediator [20]. Consistent with our results, arachidonic acid increased in the model group and decreased after ZJP intervention. Glycerol phospholipids and sphingolipids are the main structural components of cell membrane and play an important role in cell proliferation, differentiation, and apoptosis [21]. Studies have shown that LysoPC can trigger the release of arachidonic acid and promote inflammation [22]. In this study, the level of LysoPC in the MNNG group was significantly higher than that in the control group, suggesting that the disorder of the glycerol phospholipid metabolism may aggravate the occurrence and development of inflammation. After ZJP treatment, the level of LysoPC decreased significantly, suggesting that ZJP may reduce CAG by acting on the lipid metabolism. Vitamin B6 (VB6) is necessary to maintain a variety of normal metabolic levels in the body. In the process of immune response, VB6 deficiency will lead to decreasing cellular immunity, lymphoid production, and atrophy, affecting inflammatory factors [23]. In our results, the content of pyridoxamine, an important biomarker in the VB6 metabolic pathway, decreased significantly in CAG rats. It is speculated that MNNG may reduce immune function and activates inflammatory response by interfering with the VB6 metabolism. ZJP can significantly increase the expression of pyridoxamine and inhibit inflammation.

## 5. Conclusion

In this study, the changes of endogenous biomarkers in CAG induced by MNNG after treatment with ZJP were analyzed from metabolomics for the first time. Plasma and urine metabolomics provide new insights into the potential mechanism of the treatment of CAG and is of great significance to the clinical development and application of ZJP-related drugs.

## Figures and Tables

**Table 1:** Primers sequences for RT-PCR.

Genes	Forward	Reverse
TNF-*α*	ATGGGCTCCCTCTCATCAGTTCC	GCTCCTCCGCTTGGTGGTTTG
COX-2	AGGTCATCGGTGGAGAGGTGTATC	CGGCACCAGACCAAAGACTTCC
Bcl-2	TGGAGAGCGTCAACAGGGAGATG	GGTGTGCAGATGCCGGTTCAG
E-Cadherin	CCTACAATGCTGCCATCGCCTAC	GGGTAACTCTCTCGGTCCAGTCC
MCP-1	CACCTGCTGCTACTCATTCACTGG	CTTCTTTGGGACACCTGCTGCTG
IL-17A	CTGTTGCTGCTACTGAACCTGGAG	CCTCGGCGTTTGGACACACTG
*β*-Actin	GCTATTCAGGCGGTGCTGTCTC	GGCGTGTGGCAGGGCATAAC

**Table 2:** Identified metabolites of plasma and urine.

	Compound name	Formula	Mass (m/z)	R. T. (min)	KEGG	Change trend	
						MNNG/control	ZJP/MNNG
1	PC (14 : 0/18 : 1(9Z))	C40H78NO8P	730.5514	13.4216	C00157	Down	Up
2	2-Aminoacrylic acid	C3H5NO2	110.0219	15.4069	C02218	Up	Down
3	Homocysteine	C4H9NO2S	136.0426	1.6050	C00155	Up	Down
4	LysoPC (20 : 5 (5Z, 8Z, 11Z, 14Z, 17Z)/0 : 0)	C28H48NO7P	542.3325	13.4210	C04230	Up	Down
5	Prostaglandin H2	C20H32O5	375.2119	7.2675	C00427	Up	Down
6	Pyridoxamine	C8H12N2O2	169.1004	2.7954	C00534	Down	Up
7	Phytosphingosine	Pb	206.9686	1.0172	C12144	Up	Down
8	Phosphohydroxypyruvic acid	C3H5O7P	184.9860	1.8304	C03232	Up	Down
9	Serotonin	C10H12N2O	199.0824	18.3715	C00780	Down	Up

**Table 3:** Results of enrichment analysis of biomarkers.

	Pathway name	Match status	*P*	−Log (*P*)	Impact
1	Cysteine and methionine metabolism	3/33	0.000733	3.1351	0.13913
2	Arachidonic acid metabolism	2/36	0.017935	1.7463	0.09913
3	Glycerophospholipid metabolism	2/36	0.017935	1.7463	0.11182
4	Linoleic acid metabolism	1/5	0.029506	1.5301	0
5	Vitamin B6 metabolism	1/9	0.052551	1.2794	0.07843
6	Alpha-linolenic acid metabolism	1/13	0.075108	1.1243	0
7	Sphingolipid metabolism	1/21	0.1188	0.9252	0.00406
8	Glycine, serine, and threonine metabolism	1/34	0.18589	0.73075	0.02247
9	Tryptophan metabolism	1/41	0.2201	0.65739	0.10493

## Data Availability

All data are available in the manuscript, and they are shown in figures and tables.
